# Supplementary material for: Are carbon emissions trading and green financial instruments synergistic? -Comprehensive quantitative research based on content analysis
Source: PLoS One. 2024 Mar 7;19(3):e0298601. doi: 10.1371/journal.pone.0298601 (PMC10919601; doi:10.1371/journal.pone.0298601)
Supplement: S1 Data — (ZIP) [file pone.0298601.s001.zip › Data/地级市Z_F工作报告.zip!/╣≤╓▌/╣≤╤⌠╩╨/└┤╫╘╣1⁄2╓┌║┼í╛┬φ┐╦╩2╛▌═°í┐_2014.docx]

各位代表：

　　现在，我代表市人民政府，向大会作政府工作报告，请予审议，并请市政协委员、列席人员提出意见和建议。

　　一、2013年工作回顾

　　过去一年，我们在省委、省政府和市委的坚强领导下，在市人大、市政协的监督支持下，认真贯彻落实党的十八大和十八届二中、三中全会，省委十一届三次、四次全会，市委九届二次全会精神，紧扣“主基调”、实施“主战略”，坚持不懈抓好国发〔2012〕2号文件落实，围绕建设全国生态文明示范城市、在全省率先实现全面小康的总目标，按照实现“四个突破”、处理好“三个关系”的总要求，积极创新、砥砺奋进，多项指标实现历史性突破，开创了经济社会发展新局面。

　　（一）持续发力，领跑发展新速度。预计全市生产总值突破2000亿元，达2085亿元，提前实现“十二五”规划目标，占全省比重提高到26％；增速达16％，继续在全国省会城市保持前列。全社会固定资产投资3030亿元、增长22.1％。财政总收入563.8亿元、增长18.8％；公共财政预算收入277.2亿元、增长20.2％；公共财政预算支出393.49亿元、增长14.39％。金融机构存、贷款余额分别为5742.1亿元、4177.9亿元，增长30.7％、20.1％；银行业金融机构非信贷融资2427.9亿元，是2012年的2.1倍。特别令人振奋的是，在去年全省增比进位综合测评中，贵阳从2012年的第3位上升到第1位；5个区（县）位次前移，南明区跃升第一；10个区（市、县）全部进入全省第四轮经济强县建设行列。在过去一年的“竞跑”中，贵阳跑出了令人鼓舞的好成绩！

　　（二）创新驱动，激活转型新动力。大力实施创新驱动战略，综合科技进步水平指数达75.96％，上升2.8个百分点，位列全省第一；预计全年科技进步对经济增长的贡献率达55.4％。成功举办“北京·贵阳创新驱动区域合作”系列活动，全面对接北京科技、人才、资本等创新创业要素，规划建设中关村贵阳科技园，建成首都科技条件平台贵阳合作站、北京技术市场贵阳服务平台、中国技术交易所贵阳工作站。完成贵阳高校科研院所科技创新联盟、贵阳创新驱动发展战略研究院组建筹备工作；培育国家级、省级产业技术创新联盟8个，新增国家级博士后科研工作站2家，发展科技服务机构42家；引进各类高层次人才682名，建成运营贵阳国际人才城；建立天使基金、股权投资基金，成立贵阳银行科技支行、工商银行科技支行，科技创新服务体系不断健全。荣获国家知识产权示范城市称号；自主研发的人防工程管理软件荣获全军科技进步三等奖。

　　（三）结构调整，优化产业新布局。强化顶层设计，以中关村贵阳科技园为总平台，确立“一城两带六核”的产业空间布局，加快发展高新技术产业、现代制造业、现代服务业和现代农业，三次产业结构比为3.9：42：54.1。深入实施工业强市战略，扎实推进工业结构、企业规模、园区水平“三大升级计划”，京东电商产业园、国机磨料磨具等482个、总投资2256.7亿元的产业项目落户贵阳。全年实现规模以上工业增加值610.2亿元、增长20.8％，其中高新技术工业企业增加值和现代制造业增加值预计分别占36.6％、35.5％；园区工业产值占全市规模以上工业总产值的70％以上。坚持生产性和生活性服务业并重发展，预计实现服务业增加值1127亿元、增长14.6％；社会消费品零售总额785.7亿元、增长15％；金融业增加值187亿元、增长27.3％，对经济增长的贡献率达13.8％；旅游总收入728.7亿元、增长20.9％；京东贵州馆正式上线，全市电子商务交易额突破100亿元。加大农业结构调整力度、提高农业科技水平、推进农业产业化经营，特色优势农业占全部农业总产值的80％。

　　（四）建管并举，城乡呈现新面貌。深入实施城镇化带动战略，城乡一体化进程加速推进。强化规划引领，按照都市功能核心区、都市功能发展区、城市发展拓展区、生态保护发展区“四个功能分区”推进城市组团式发展。完善体制机制，构建城市可持续开发建设新模式，促进房地产业健康发展。加大交通基础设施建设力度，全面建成“三环十六射”骨干路网；轨道交通1号线全面开工；加快推进省际高铁、市域快铁和跨区域高速公路建设；龙洞堡国际机场2号航站楼建成投用，年旅客吞吐量突破千万人次；完成中心城区畅通工程二期规划编制；成功申报“全国公交都市”试点城市。扎实开展“一创四办”活动，加强市容环境卫生管理；成功入选住建部2013年度国家智慧城市试点，建成并试运行数字化城市管理二期工程，不断完善水、电、气等城市基本生活要素保障机制。以“5个100工程”为载体，带动城乡建设和产业发展，入选全省的70个项目累计完成投资1084.9亿元。开工改造棚户区城中村项目42个、474万平方米。实施村级综合楼改造提升工程，新建81栋、维修437栋、改造789栋。建成通村油路（水泥路）450公里，行政村油路（水泥路）通畅率91.8％，完成基本农田建设3.3万亩，新增、改善和恢复灌溉面积11.2万亩，建成美丽乡村示范点27个，城乡发展基础不断夯实。

　　（五）生态优先，建设宜居新家园。严格执行《贵阳市建设生态文明城市条例》，大力实施“护林、治水、净气、保土”工程，强力推进“创模”攻坚。建成小车河城市湿地公园二期，改造提升花溪十里河滩国家城市湿地公园和黔灵山公园；实施石漠化治理17.2万亩，完成营造林24万亩，新增绿地103万平方米，森林覆盖率和建成区绿化覆盖率分别达44.2％、43.5％；加强水源保护和河流治理，继续实施“两湖一库”“五大治理工程”，初步完成南明河水环境综合治理一期工程，集中式饮用水源地水质逐年向好；开展空气质量治理“七大攻坚工程”，空气质量优良率达76.2％（2013年国家新标准）；大力推进节能减排和再生资源利用，淘汰落后产能158.2万吨，可再生能源建筑应用272万平方米。生态文明贵阳会议升格为生态文明贵阳国际论坛，并成功举办2013年年会。

　　（六）高端引领，构建开放新格局。贵阳综合保税区获批设立，成为全省首个综合保税区，正全力加快建设，着力打造国际化开放大平台。依托龙洞堡国际机场，启动贵州双龙临空经济区规划编制等前期工作。成立驻京投资促进局，选派77名干部赴京挂职。全面加强招商引资工作，引进省外到位资金1666亿元、增长39％，实际直接利用外资6.3亿美元、增长32.9％，外贸进出口总额预计完成63.2亿美元、增长25.1％。开展“营改增”试点，推进工商登记制度便利化，全市私营企业、个体工商户注册数分别增长29.2％、18.6％，企业和个体工商户注册资本达3212.9亿元，非公有制经济占生产总值比重突破50％，改革开放红利逐步释放。

　　（七）以人为本，增进民生新福祉。城镇居民人均可支配收入23376元、农民人均纯收入9592元，分别增长10％、13％。全面完成“十件实事”年度目标，民生投入占市级财政支出的61.1％；云岩、南明、白云、观山湖在全省同步小康创建活动中获通报表彰。新增城镇就业20.3万人，城镇登记失业率控制在4％以内。城镇职工基本养老、医疗、失业、工伤、生育保险征缴扩面；13.1万城乡低收入人口纳入最低生活保障。新开工建设公共租赁住房17455套、竣工2806套；完成农村危房改造5000户。102个贫困村实现“减贫摘帽”，荣获全省“整市脱贫特别奖”。新建平价生鲜直销店100个、平价猪肉直销店50个；居民消费价格较上年上涨3.2％。

　　（八）全面发展，书写和谐新画卷。扎实推进教育“9+3”计划，教育投入占生产总值比重达4.2％；全面完成学前教育三年行动计划，乡镇公办中心幼儿园实现全覆盖；入选国家中小学教育质量综合评价改革试验区；贵阳职业技术学院、贵阳护理职业学院被评为省级示范高职院校。建成覆盖城乡的三级医疗卫生网络，国家基本药物制度实现全覆盖，减免城乡低收入和低保对象医疗费用3834.5万元，惠及12.98万人次。人口出生率、人口自然增长率分别为9.96％。、4.85％。，全面完成计生工作“双降”目标。成功举办中超、亚冠等重大体育赛事；获批创建国家公共文化服务体系示范区，建成10万户农村直播卫星工程。巩固城市基层管理体制改革成果，“一核多元”社会共治模式获“中国城市管理进步奖”；启动农村社会治理“网底工程”试点工作。加强流动人口管理和服务，为75.7万人办理“城市绿卡”，提高同城化待遇，被列为国家流动人口卫生和计生基本公共服务均等化试点城市。严格落实安全生产责任制，安全生产事故起数与死亡人数保持“双降”；深入开展“两严一降”专项行动，刑事发案数下降18.2％，群众安全感达94.2％，比上年提高4.8个百分点，第三次荣获全国社会治安综合治理奖“长安杯”。加强群众工作，信访积案化解率达96.1％。同时，国防、国家安全、司法行政、民族宗教、移民、史志、档案、外事、侨务、对台、气象、老龄、食品药品安全、工青妇、关心下一代、慈善、残疾人、红十字、爱国卫生等工作全面发展。

　　（九）转变作风，提升服务新效能。以“找问题、找差距、找目标”主题大讨论活动为抓手，切实加强政府自身建设，全面提高工作效能。严格执行中央“八项规定”、省委“十项规定”及市委29条实施细则，坚决反对“四风”，压缩行政经费8％用于教育支出。加大反腐力度，查处违纪违法案件226件，给予党纪政纪处分275人。加强法制建设，制定政府规章21件，受理行政复议147件。办理人大代表建议和政协提案489件，回复率达100％。进一步简政放权，市级行政审批项目减少到88项，办理时限压缩50％以上。在2013年中国社科院发布的《中国政府透明度年度报告》中，贵阳位列第四。

　　各位代表！回顾一年的工作，让我们最振奋的是创新驱动强势起步，让我们最自豪的是城乡面貌日新月异，让我们最欣慰的是发展成果全民共享！这些成绩的取得，是省委、省政府和市委正确领导的结果，是市人大、市政协有效监督、大力支持、全程参与的结果，是全市干部群众共同奋斗的结果。同时，也凝聚了已调离贵阳市的李军书记、李再勇市长的心血、智慧和汗水。在此，我代表市人民政府，向全市各族干部群众，向各位人大代表和政协委员，向各民主党派、工商联、无党派人士、各人民团体和各界人士，向驻筑人民解放军、武警官兵和人民警察，向中央及省在筑单位，以及所有关心、支持贵阳建设发展的各位老领导、老同志和各界朋友，向李军书记、李再勇市长表示衷心的感谢，并致以崇高的敬意!

　　成绩催人奋进，问题不容忽视。通过开展“找问题、找差距、找目标”主题大讨论活动，我们清醒地看到，贵阳欠发达的基本市情没有改变，发展过程中还存在不少问题：在经济发展方面，经济总量小、人均水平低，全市生产总值、人均生产总值分别为西部省会城市平均水平的67％、47.1％；经济结构不优、实体经济不强、转型升级任务重，虽然形成“三二一”的产业结构，但高新技术产业、现代制造业、现代服务业、现代农业占比不高，生产性服务业发展滞后；科技投入不足，全社会研究与试验发展经费仅占生产总值的1.35％，低于全国平均水平；投资结构不优、综合效益偏低，工业投资仅占全社会固定资产投资的25.3％。在社会管理方面，社会治安形势虽然通过开展“两严一降”专项行动有所好转，但离老百姓的期望和建设全国生态文明示范城市的要求还有较大差距；安全生产形势不容乐观，有的隐患排查不彻底、台账不清、责任落实不到位，个别重点行业领域事故仍然多发。在城市规划建设管理方面，“疏老城、建新城”的推进速度较慢，城市开发模式和土地供应方式较粗放，规划执行不严，违法违章建筑多，公共绿地少，“脏、乱、差”现象回潮反弹，交通拥堵，行车难、停车难等问题较为突出。在民生保障方面，虽然持续加大民生投入，但由于经济欠发达、历史“欠账”多，涉及老百姓切身利益的收入低，就业难，上学难、上好学难，看病难、看病贵，养老难，住房难，买菜难、买菜贵等民生问题仍然突出，老百姓的意见较大。在生态建设和环境保护方面，环境压力不断加大，特别是大气环境方面，由于燃煤、扬尘、机动车尾气排放等原因，空气在部分时段出现PM2.5超标现象，有污染情况。水环境方面，“两湖一库”、花溪水库等“城市水缸”还存在内源和外源污染隐患；地表水功能区范围内污水收集管网覆盖不全，老城区雨污混流，存在污水直排现象。在政府自身建设方面，统筹力、协调力、执行力、落实力、担当力与“作表率、走前列”，当好“火车头”、“发动机”的要求还不相适应，政府治理体系和治理能力还需要进一步加强，破解发展难题、应对复杂局面的能力还需要进一步提升。针对这些问题，我们将在下一步工作中采取有效措施，切实加以解决。

　　二、2014年工作安排

　　今年是深入贯彻落实党的十八届三中全会精神，全面深化改革扩大开放的第一年，既是改革之年、开放之年、创新之年，又是执行之年、攻坚之年、突破之年。做好全年工作，意义十分重大。

　　今年经济社会发展的总体要求是：以邓小平理论、“三个代表”重要思想和科学发展观为指导，全面贯彻落实党的十八大和十八届二中、三中全会，中央经济工作会，省委十一届四次全会、全省经济工作会，市委九届三次全会精神，在省委、省政府和市委的坚强领导下，坚持稳中求进、提速转型、又好又快的总基调，坚持主题主线和主基调主战略，坚持创新驱动、聚焦转型升级，按照“紧扣一个目标、守住两条底线、强化三种意识、狠抓四个关键”的要求，紧紧依靠“三大动力”、扎实推进“六项战略任务”，奋力走出一条西部欠发达城市经济发展与生态改善“双赢”的可持续发展之路，全力打造贵阳发展升级版，加快建设全国生态文明示范城市，切实当好全省的“火车头”和“发动机”。

　　今年经济社会发展的主要预期目标是：生产总值增长16％；全社会固定资产投资增长15％；社会消费品零售总额增长15％；引进省外到位资金增长30％；实际直接利用外资增长20％；外贸进出口总额增长20％；公共财政预算收入增长18％；城镇居民人均可支配收入增长14％，农村居民人均可支配收入增长15％；城镇新增就业21万人，城镇登记失业率控制在4％以内；人口自然增长率控制在5％。以内；居民消费价格指数控制在省调控目标范围内；万元生产总值综合能耗、主要污染物排放总量均控制在省下达目标范围内；城镇化率提高1.6个百分点；森林覆盖率达45％以上；空气质量优良率达80％以上。

　　各位代表，市委九届三次全会吹响了我市全面深化改革扩大开放的冲锋号，发出了全力打造贵阳发展升级版的动员令。今年，我们将紧紧围绕“改革、开放、创新、转型、投资、城镇、生态、民生”八个重点，努力实现八大升级，聚力打造贵阳发展升级版。

　　（一）改革——我们的最大红利，要与时俱进、攻坚克难，以涉险滩、破藩篱的勇气奏响改革最强音，推进发展动力升级

　　按照“突出问题导向、体现贵阳特色、承担历史责任”的要求，坚持以开放倒逼改革、以问题倒逼改革、以需求倒逼改革、以目标倒逼改革，充分发挥市场对资源配置的决定性作用，敢于打破陈规，冲破思想障碍，突破利益固化，在全省深化改革中先行先试、领先领跑。

　　一是紧紧围绕转型升级深化经济领域改革。在发展民营经济方面，以消除各种隐性壁垒和营造稳定、透明、公平的市场环境为重点，积极探索“负面清单”管理模式，实行统一的准入制度；支持鼓励民营企业进入特许经营领域；启动实施提高民营经济比重五年行动计划。在国企改革方面，选择部分行业和国有企业开展混合所有制经济试点；完善国有资产管理体制,以管资本为主加强国有资产监管，成立市属国有企业产业发展基金，支持有条件的国有企业改组为国有资本投资公司；探索建立职业经理人制度。在推进军民融合方面，建立贵阳军民结合产业发展研究机构或产业联盟，制定军民融合发展政策，鼓励地方企业与军工企业兼并重组或相互参股控股，支持军工人才创办、领办民营科技型企业。在财税金融改革方面，深化预算管理改革，实施全面规范、公开透明的预算制度，探索建立跨年度预算平衡机制和全过程预算绩效管理体系；按照国家统一部署，进一步扩大“营改增”试点范围，做好地方税体系建设工作；制定政府性债务管理办法，建立风险预警机制和偿债准备金制度；改组成立市金融投资控股公司；推进投融资平台公司分类管理，向实体化发展。在农村产权制度改革方面，全面完成农村土地承包经营权确权登记颁证，启动农村果权、茶权确权登记颁证试点工作；建立市级农村产权交易公共平台，健全县、乡、村土地流转服务网络；推进农村小型水利设施产权制度改革试点工作；改革完善农村宅基地制度，积极争取开展农民住房财产权抵押、担保、转让试点工作，探索增加农民财产性收入渠道，推进城乡要素平等交换和公共资源均衡配置，让农民平等参与现代化进程、共同分享现代化成果。

　　二是紧紧围绕转变政府职能深化行政体制改革。完成市、县两级政府机构改革，积极推进县（市）改区、乡改镇工作，稳妥推进事业单位分类改革；研究制定政府各部门权力清单。完善市政府重大行政决策程序规定。清理、分批取消和下放投资项目审批、生产经营活动和资质资格许可等事项，取消一批市场和社会能自我调节的审批事项，进一步下放一批区县政府能够履行的职能事项；严格控制新增审批项目，凡没有法律法规和规章依据的，一律不得设定或变相设定行政审批项目。完善政府向社会组织购买服务机制，推行政府向社会组织委托职能和购买服务目录公示制度。科学设置工作考核和政绩考评指标体系，制定差异化考核标准和办法，实行分类考核。

　　三是紧紧围绕提升城市软实力深化文化体制改革。培育和践行社会主义核心价值观，大力弘扬“知行合一、协力争先”的城市精神，全力提振贵阳发展自信。推动文化行政管理由办文化向管文化、由管微观向管宏观转变，加快形成管人管事管资产管导向相统一的国有文化资产管理体制。深化文化市场综合执法改革，构建健康有序的城乡文化市场管理机制。整合新闻媒体资源，提升传统媒体和新兴媒体融合发展水平。

　　四是紧紧围绕提升环境质量深化生态文明体制改革。开展建立空间规划体系试点工作。对全市水流、森林、湿地、山岭、荒地、生物多样性等进行普查登记。开展自然资源资产价值评估、自然资源资产产权制度和用途管制制度试点。完善应对生态环境变化预测预警和应急处置机制。制定环境污染第三方治理实施细则，在开磷集团等企业开展第三方治理试点。启动水、空气环境质量及污染源自动监测系统建设，建立监测数据利用制度。坚持谁受益、谁补偿的原则，完善对“两湖一库”等重点生态功能区的生态补偿机制，加大财政转移支付力度。建立水资源有偿使用制度，水资源费使用向重要饮用水源保护地倾斜。

　　五是紧紧围绕保障和改善民生深化社会体制改革。在社会保障方面，完善最低生活保障、就业困难群体就业援助、重特大疾病保障和救助等制度；健全并落实城乡最低生活保障标准与物价涨幅挂钩联动机制；开展城乡居民大病保险试点；制定完善保障性住房准入、使用、退出等机制。在教育方面，实施教育立市战略，全面推进教育布局调整、资源整合利用、教育管理体制、办学体制、人事和分配制度、人才培养模式、招生考试、民办教育、开放合作办学、教育信息化等方面改革。在养老服务方面，大力发展社区养老、助老服务，探索向社会组织购买社区养老服务，加快建立社会养老服务体系。在卫生计生方面，抓好全科医生执业方式和服务模式改革国家试点工作；按程序启动实施单独两孩生育政策。在户籍管理方面，推进农业转移人口市民化改革，研究制定全域贵阳城乡居民自由迁徙服务管理办法。在社会治理方面，鼓励和支持区（市、县）建立社会组织孵化基地；健全和完善社区社会组织登记备案制度；实现行业协会商会与行政机关彻底脱钩；推进诉访分离改革。

　　（二）开放——我们的战略引擎，要构筑平台、拓宽领域，以开放新格局打造发展新高地，推进经济外向度升级

　　充分利用国际国内两种资源、两个市场，从开放的高度、广度、深度着力，启动实施外贸外资外经“三外”翻番计划，提升经济发展的外向度。

　　一是突出“软硬并行”，搭建对外开放新平台。办好酒博会、茶博会等重要展会活动，探索建立智能化“电子口岸”，全力打造开放“软平台”。围绕打造综合保税区、临空经济区对外开放“双引擎”，加快推进综合保税区一期围网工程建设，确保今年9月份顺利通过验收，年底封关运行，在中瑞自由贸易（协定）框架内，积极加强与瑞士的经贸交流合作；充分发挥空港优势，按照“坚持一个定位、狠抓两个关键、突出三个创新、处理四个关系”的要求，加快推进贵州双龙临空经济区规划建设工作，集中精力发展与航空关联度高的现代服务业；启动建设贵阳无水港，全力搭建开放“硬平台”。

　　二是突出“内外并举”，构建对外开放新格局。牢牢把握与北京开展区域合作的重大机遇，主动融入长江经济带，深化与京津冀、长三角、珠三角、成渝、北部湾、长株潭等区域的交流合作，努力推进产业互动、资源互利、市场共享；围绕黔中经济区建设，积极与贵安新区及周边市（州）联动，推进优势互补、一体化发展。扩大与港澳台、东盟、欧美的经贸往来和文化交流，进一步扩大外贸进出口规模。密切与国内外友好城市（城区）的交流，扩大合作成果。

　　三是突出“招大引强”，扩大对外开放新成效。围绕产业重点、盯住产业链条，做深项目前期工作、创新招商引资方式、优化投资服务环境，提升招商水平和质量，全力打造内资外资“抢滩登陆”的投资高地。充分发挥中关村贵阳科技园、综合保税区、临空经济区、“5个100工程”等平台载体作用，力争引进10家世界500强或国内500强企业；引进省外到位资金2200亿元，产业类项目占40％以上；确保实际直接利用外资7.6亿美元。

　　（三）创新——我们的不竭动力，要内外联动、高位对接，以科技创新引领跨越发展，推进核心竞争力升级

　　以科技成果转化和产业化为核心，构建创新创业生态系统，提升科技创新能力，打响贵阳“智”造品牌。

　　一是建强创新平台。举全市之力推进中关村贵阳科技园建设，加快推进贵州科学城、中科院贵州科技创新园、国家级矿产资源综合利用工程技术研发中心、中央商务区高端写字楼等项目和基础设施建设，迅速把中关村贵阳科技园的骨架立起来、环境做出来，确保中关村贵阳科技园核心园精彩亮相。精心筹办“北京·贵州云计算及大数据合作洽谈会”，确保引进移动互联网、云计算、大数据等战略性、先导性项目100个；全面动工（启动）“北京·贵阳创新驱动区域合作”系列活动签约项目，年内完成投资60亿元以上，着力打造一批标志性园区、标志性产业、标志性企业及标志性技术，推进中关村贵阳科技园建设取得突破性成效。

　　二是优化创新环境。按照“简、优、限”的要求，提升政务服务效能；坚决兑现各项创新创业优惠政策，着力营造诚信的政务环境；积极弘扬创新精神和培育创新文化，鼓励创新、宽容失败，在全社会形成创新成果共享、创新风险共担的良好氛围；加强知识产权运用和保护，依法严厉打击侵犯知识产权的违法犯罪行为，努力营造公平、开放、有序的市场环境。

　　三是聚集创新要素。紧扣人才、科技、金融三大创新核心要素，以建设国家创新型试点城市为载体，大力实施“黔归人才计划”、“科技人才工程”、“企业家培育工程”，充分发挥中关村贵阳科技园青联凝聚青年人才、服务创新发展的作用，加快推进高技能人才培训基地以及高级人才公寓等配套设施建设，坚持省市联动、高标准提升贵阳国际人才城，打造人才创业首选地的重要支撑平台，引智天下、筑梦贵阳；大力实施“科技服务工程”，充分发挥“一站一台”、中国技术交易所贵阳工作站作用，提升科技咨询和技术市场交易服务，促进技术交易和成果转化；大力实施“科技金融工程”，支持和引导金融机构设立服务科技型企业的专营机构，建立科技担保、科技信贷、创业投资等科技金融服务体系，积极破解创新发展融资难题。

　　四是提升创新能力。把握“大数据时代”特征，积极申报国家下一代互联网示范城市，抢抓中国电信、移动、联通三大运营商落户贵安新区的机遇，大力发展大数据产业、新一代信息技术产业；承办好2014年“中国（北京）跨国技术转移大会”贵阳分会和中国电子商务创新发展峰会；依托贵阳高校科研院所科技创新联盟、贵阳创新驱动发展战略研究院等组织机构，充分激发企业、高校和科研院所的创新热情，提升核心技术研发和科技成果产业化转化能力。力争新增龙头型高新技术企业20家以上，专利申请量、专利授权量分别增长20％以上，全社会研究与试验发展经费占生产总值比重达1.7％以上，科技进步贡献率达56％以上。

　　（四）转型——我们的必由之路，要瞄准高端产业和产业高端，以转方式、调结构壮大实体经济，推进产业升级

　　树立产业强则城市强、产业有竞争力则城市有竞争力的理念，坚持“快、转、高”并举，把产业转型升级作为加快发展的主攻方向，积极推进产业结构调整，加快构建核心竞争力强、规模效益突出的现代产业体系，推进全市产业从“追赶型”向“引领型”转变发展。

　　一是以高新技术产业和现代制造业为龙头加快发展现代工业。切实强化高新技术产业和现代制造业对实体经济的引领带动作用，着力构建以高新技术产业为先导、现代制造业为支撑的现代工业体系，确保规模以上工业增加值达730亿元，高新技术产业增加值、现代制造业增加值均增长30％以上。抓好产业集聚区建设。把中关村贵阳科技园作为工业强市的主战场，大力实施园区引领、科技创新、科技金融、配套环境提升、创新创业人才聚集和千亿产业百亿企业培育“六大工程”，推进产业园区化、园区集聚化发展，加快把信息、新材料、装备制造、生物医药打造成千亿级产业集群。严格执行中关村贵阳科技园分园认定程序及入园标准，所有新引进工业项目全部分类入园发展。抓好传统产业改造升级。大力运用高新技术、先进适用技术和信息技术改造提升一批传统资源型产业，实施首钢贵钢新特材料、开磷息烽煤电磷煤电化一体化、紫江水泥磷渣微粉等一批技改项目，启动贵铝氧化铝厂搬迁工作，推进“四个一体化”取得重大突破，进一步延伸产业链末端、抢占价值链高端，资源就地转化率达85％以上。抓好落后产能淘汰和过剩产能化解。严格执行行业专项政策和准入条件，禁止引进能耗高、污染大、效益低的项目，杜绝低水平重复建设。淘汰落后产能78万吨。

　　二是以金融业为龙头加快发展现代服务业。切实强化现代服务业对实体经济的支撑作用，着力构建以金融业为龙头，生产性服务业和生活性服务业并重、现代服务业和传统服务业并举的服务业体系，进一步丰富消费业态、培育消费热点、优化消费环境，促进服务业结构提质、发展提速、比重提高、水平提升。确保服务业增加值增长14.5％，其中现代服务业增加值占比达49％。做强金融业。围绕打造区域性金融中心和西部科技金融创新城市，深入实施“引金入筑”工程，新引进金融机构2家，设立科技银行2家；加快推进贵州金融城建设，力争建成40万平方米，确保贵阳农商行总部大楼竣工。积极推进企业信用培育、科技信贷创新、银企交流公共服务平台搭建，探索培育贸易金融、网络金融、消费金融等业态，支持天使投资、创业投资和各类私募股权基金发展，扶持一批企业上市融资和在“新三板”挂牌，增强金融运行效率和服务实体经济能力。金融机构存、贷款余额分别增长20％、19％，银行业金融机构非信贷融资增长20％。做大信息服务业。积极争取工信部和中国电信、移动、联通三大运营商支持，推进贵安新区数据中心上联电路从贵阳市中转，力争成为第三批国家级互联网骨干直联点；深入推进国家智慧城市建设试点工作，加快信息基础设施建设，强化信息技术支撑和应用，发展壮大信息服务业。做特旅游会展业。扎实推进旅游环境提升、旅游功能提升等六大升级计划，加快青岩古镇、天河潭、桃源河、南江大峡谷等旅游景区改造提升，推进南明河城市流域休闲度假带、多彩贵州城等建设，精心筹办好全省第九届旅游发展大会；积极做好入境旅客落地签证服务。全市旅游接待总人数和旅游总收入均增长20％以上。做好2015年比利时布鲁塞尔国际烈性酒大赛筹备工作，办好书博会、制博会、亚洲青年动漫大赛等重大会展活动，实现会展综合经济效益120亿元以上。做活商贸物流业。进一步优化商业街区、零售网点、大型专业批发市场、物流园区布局，抓好京东电商产业园建设和京东贵州馆运营，打造新兴电商“网城”；推进西南国际商贸城、普天现代物流创业园、“马上到”云服务铁路港等项目建设，加快建立社会化、专业化现代商贸物流服务体系。同时，大力发展总部经济、文化创意、研发设计等生产性服务业和健康养老、餐饮住宿、家政服务等生活性服务业。

　　三是以都市农业为龙头加快发展现代农业。坚持以“三化”兴“三农”，用现代产业体系提升农业、现代经营形式推进农业、现代科学技术改造农业，把现代农业建成农民的绿色银行、市民的绿色游园、城市的绿色屏障，让农业成为有奔头的产业，让农民成为体面的职业。确保一产增加值增长6％以上，其中现代农业占比达26％。优化农业产业结构。按照生态产业化、产业生态化的理念和规模化、特色化、都市化的要求，进一步调整农业种植结构和品种结构，集中力量做大做强蔬菜、茶叶、水果、中药以及畜禽等特色优势产业。推进农业产业化经营。鼓励有条件的农户流转承包土地经营权，发展多种形式规模经营。进一步拓宽农业综合功能，推进农业与食品加工、旅游休闲、健康养生等产业融合发展。继续扶持农业龙头企业、培育农业优质品牌，壮大新型农业经营主体，新发展农产品加工企业30家、农民专业合作社100个、家庭农场100个，农产品加工转化率提高2个以上百分点。充分利用淘宝贵州馆、京东贵州馆大力推销本地农副产品，拓宽销售渠道。强化农业科技支撑。依托贵阳生态农业科技创新区建设，加快构建农业科技创新体系，在广泛应用新型农业实用技术的同时，实施一批现代农业科技项目，提高耕地产出率和农产品附加值。健全农业服务体系。扶持发展农民用水合作组织、农业专业技术协会、农民经纪人队伍，支持具有资质的经营性服务组织从事农业公益性服务，积极推行合作式、订单式、托管式服务模式，提高农业社会化服务水平。

　　（五）投资——我们的重要支撑，要优化结构、提升质量，以有效益的投资确保无水分的增长，推进发展增量升级

　　坚持创新驱动与投资拉动相结合，以项目为主抓手、以实体经济为主方向，扩大有效投资、保持合理规模，以优质增量支撑经济强劲增长。

　　一是紧盯重点项目，推进投资快速增长。落实好“储备一批、申报一批、开工一批、建成一批”的良性滚动机制，以中关村贵阳科技园、“5个100工程”、191个省重大工程和重点项目为重点，通过大项目、大工程的支撑带动，保持投资强劲增长势头。确保全年完成全社会固定资产投资3485亿元，其中省重大工程和重点项目完成投资1077亿元。

　　二是优化投资结构，提高投资综合效益。加大产业项目招商引资力度，进一步发挥财政扶持资金、产业发展资金、各类发展基金的引导作用，撬动更多社会投资向高新技术产业、现代制造业等实体经济聚集，着力优化投资结构，通过有效益的投资和无水分的增长，从源头上推动产业转型升级。力争产业投资占全社会固定资产投资的40％以上。

　　三是强化要素保障，加快项目建设进度。按照“六规合一”的要求，做深、做实项目前期准备工作，确保投资项目库动态保持在1万亿元以上。组建由市级领导负责的重点工作和重大项目指挥部，深入开展进项目、进企业、进工地“三进”服务活动，千方百计保障资金、土地、用工、运输及煤、电、油、气、讯、水等要素，努力破解融资难、征地难、用工难等瓶颈难题，全面提升项目履约率、落地率、资金到位率、投产达产率，形成大干快上、持续发力的投资热潮。

　　（六）城镇——我们的共同家园，要以人为核心、健康发展，以新型城镇化带动城乡一体化，推进城镇品质升级

　　按照“疏老城、建新城”的总要求和“生产空间集约高效、生活空间宜居适度、生态空间山清水秀”的总布局，积极推进以人为核心的新型城镇化，促进城乡一体化发展。

　　一是进一步优化城市空间布局。依托现有山水脉络，严格按照都市功能核心区、都市功能发展区、城市发展拓展区、生态保护发展区“四个功能分区”，进一步完善城市总体规划和城市功能设计，合理控制城市组团增长边界，引导城市布局从单极向多极转变，构建以老城区、观山湖区为核心的“两核多组团”城市空间布局。进一步强化规划引领和规划执行，突出城市生态特色，保护城市历史文化，让城市融入自然，让市民留住记忆。

　　二是进一步优化城市开发模式。严格执行促进房地产业持续健康发展系列配套文件，实施“净地”出让，坚决把土地一级市场掌控在政府手里，充分运用土地级差地租、市场竞争机制，提高城镇建设用地利用效率，促进土地市场供需平衡，推进房地产业健康可持续发展。优化资源配置，鼓励发展工业、旅游和物流等复合地产。

　　三是进一步优化城市交通体系。按照“联通外循环、打通内循环、畅通微循环”的要求，全面实施中心城区畅通工程二期，启动1.5环、快速公交（BRT）、连接线和打通“断头路”等重点工程，加快建设轨道交通1号线、开工建设2号线，着力优化中心城区路网结构；充分发挥交通智能监控指挥系统作用，提升交通管理组织能力，下大力气治理交通拥堵。主动融入全省高速公路、铁路、水运建设“三年会战”，加快建设贵阳至黔西高速公路、北京东路延伸段二期，市域快铁，贵广、长昆、渝黔、成贵省际高铁；建成白云至修文、观山湖至扎佐城市干道，厦蓉高速清镇至织金段，确保贵广高铁、市域快铁贵开线、久永线建成通车，满怀激情迎接“高铁时代”的到来！

　　四是进一步优化城市宜居空间。通过“疏密结合、肥瘦搭配、项目捆绑、异地平衡、统筹还贷”的方式，探索建立老城改造与新区建设联动平衡机制，加快“疏老城、建新城”。在疏老城方面，按照“降低人口密度、降低建筑密度、增加公共绿地、增加公共空间”的要求，坚持“多拆少建”、“只拆不建”，下决心用三年时间完成中心城区非成片棚户区改造，今年启动项目20个；五年内完成中心城区集中成片棚户区、“三线”企业棚户区改造，今年分别开工建设项目38个、8个。在建新城方面，联动老城改造，推动优质教育、医疗、文化、体育等公共资源和专业市场、商业综合体向新区布局，引导中心城区人口有序向一环外疏解，提升城市宜居度。

　　五是进一步优化城市环境管理。巩固创文、创卫成果，着力建立市容市貌、环境卫生长效管理机制，针对“脏、乱、差”及一些不文明行为，深入开展“五脏五乱”专项整治行动，让“爽爽的贵阳”更加文明整洁。健全完善城市供水、供电、供气、气象灾害等应急反应机制和预警预告制度，提高快速反应和应急处置能力，提升城市科学化、精细化、长效化管理和服务水平。

　　六是进一步优化城乡基础条件。以“四在农家·美丽乡村”基础设施建设六项行动计划为重点，建成一批小康路、小康水、小康房、小康电、小康讯、小康寨；完成开阳白安河水库、乌当金家箐水库、息烽廖九寨水库等骨干水源水库主体工程；建成“美丽乡村·幸福家园”示范点40个，继续实施村级综合楼改造提升工程，推动城市基础设施和公共设施向农村延伸。以实施“5个100工程”为抓手，推进一批承载力强、配套率高、聚集效应明显的产业园区，建成一批基础设施配套、经营机制完善、综合效益明显的现代高效农业示范园区，发展一批有历史记忆、地域特色、民族特点的示范小城镇，打造一批环境优美、功能复合、带动力强的城市综合体，提升一批知名度高、影响力大、资源禀赋突出的旅游景区，以产城互动推进城乡互动、一体化发展。

　　（七）生态——我们的最大优势，要坚守底线、倍加珍惜，以生态文明理念开创绿色发展未来，推进宜居环境升级

　　深入实施《贵阳建设全国生态文明示范城市规划》，认真落实全省环境保护十二件实事，扎实推进“创模”攻坚，提高环境质量，让贵阳的天更蓝、地更绿、水更清、气更净。

　　一是践行生态文明理念。坚持尊重自然、顺应自然、保护自然，将生态文明理念贯穿到经济社会发展的各方面和全过程。在省服务保障工作领导小组的领导下，办好生态文明贵阳国际论坛2014年年会，加快永久会址建设，加强论坛成果转化，打造传播生态文明理念的中国窗口、扩大贵州对外开放的高端平台。扎实开展全国节能减排财政政策综合示范城市建设，大力推进工业低碳化、交通清洁化、建筑绿色化、主要污染物减量化、可再生能源利用规模化。深入开展生态文明“八进”活动、“绿丝带”志愿者活动和绿色社区创建活动，广泛普及生态文化，大力倡导低碳生活和消费方式，践行绿色、循环、低碳发展。

　　二是加大生态执法力度。坚决执行《贵阳市建设生态文明城市条例》，强化生态法制宣传教育，充分发挥生态文明委和“两湖一库”管理局、生态保护公安分局等职能作用，大力支持生态保护审判庭和法庭、生态保护检察局工作、生态保护公安分局等职能作用，从源头上入手、严把项目准入关，在巡查上下力、严把污染监督关，坚决取缔和淘汰落后产能，严惩重罚破坏生态环境的行为，以更加强硬的态度、更加严厉的措施保护生态环境。

　　三是优化生态宜居环境。深入实施“蓝天守护计划”，编制实施《贵阳大气污染防治规划》，逐步畅通城市200米以下通风走廊；推进无煤城市建设，确保年内完成老城区管道天然气置换人工煤气工程，规范城区燃煤使用管理，并有序推进取缔工作；加强建筑施工工地、道路扬尘污染防治以及机动车尾气、饮食油烟污染等综合治理，实现空气质量自动监测站县城全覆盖，努力保持贵阳空气清新。深入实施“碧水治理计划”，加强“两湖一库”等重要水源地保护与治理，积极探索跨区域联动调水机制，全力保障城市供水安全稳定；建成地表水环境质量自动监测系统；加大污水治理力度，推进南明河水环境综合治理二期工程，加快建设城市雨污分流管网，完成5条支流综合治理，建成污水处理厂4座、改造提升5座；巩固国家节水型城市创建成果，大力倡导节约用水。深入实施“绿地保卫计划”，完成花溪十里河滩国家城市湿地公园二期改造提升工程；推进红枫湖、百花湖、红枫湖、小关湖湿地公园建设；新增营造林20万亩、绿地300万平方米，实施环城绿化1.5万亩；大力实施环城林带保护工程、龙洞堡片区生态修复工程、资源开采区修复工程和城市绿化景观提升工程，让贵阳市民拥有绿环、绿带、绿轴、绿核相连接的绿色生态空间。

　　四是创新生态制度体系。健全生态文明建设评价指标体系和考核机制，加强生态环境法治建设。建立生态环境监测信息发布制度，公开环境质量、环境管理、企业环境行为等信息，维护市民的环境知情权、参与权和监督权，拓宽社会参与生态保护的渠道，形成人人关心环境、爱护环境、共享环境的浓厚氛围。建立公众参与第三方监督制度，健全环境损害赔偿制度，探索建立生态环境损害责任终身追究制度，坚决不碰“生态红线”，坚决严守“生态底线”。

　　（八）民生——我们的为政之基，要以人为本、协调发展，以改善民生为重点促进社会和谐，推进市民幸福指数升级

　　在集中力量办好“十件民生实事”的同时，紧紧围绕“富民、安民、利民、便民”的目标，把就业作为最基本的民生、教育作为最长远的民生、医疗作为最现实的民生，努力使发展成果更多更公平地惠及城乡居民，让我们的老百姓生活得更好、更幸福！

　　一是谋富民之策。做好高校毕业生、农村转移劳动力、下岗失业人员和“零就业家庭”的就业工作，全面落实“3个20万”、就业服务补贴、返乡创业等优惠政策，促进充分就业、就近就业。深入实施“城乡居民增收工程”，拓宽增收渠道，增加城市居民工资性收入和农村居民财产性收入，确保城乡居民收入稳定增长。坚持脱贫不脱钩，构建专项扶贫、社会扶贫、行业扶贫“三位一体”的精准扶贫开发体系，持续改善群众生活条件。

　　二是尽安民之责。坚持保障安民。以高于10％的增长比例全面提高城乡居民最低生活保障标准；城镇居民基本医疗保险和新型农村合作医疗政府补助标准提高到320元，新农合统筹基金最高支付限额提高到15万元；稳步推动城乡养老保险转移接续；加快保障性住房建设，新开工公共租赁住房17万平方米、竣工10万平方米，构建以政府为主提供基本保障、以市场为主满足多层次需求的住房保障供应体系。坚持和谐安民。强化社会治理体系和治理能力建设，在加强社区建设管理的同时，切实解决好居委会运行中存在的突出困难，建强维护社会稳定的第一道防线。加强流动人口管理，流动人口登记率、出租屋登记率均达90％以上。按照“十个一”的新要求，深入开展“两严一降”工作，扎实推进社会治安综合治理，健全“点、线、面”相结合的社会治安立体防控体系，确保刑事发案数下降20％，群众安全感保持在90％以上；强化安全生产责任制，深化隐患排查、专项治理和“打非治违”，坚决遏制各类重特大事故发生；进一步健全完善突发事件应急处置机制，提高应急处置能力和水平；严格执行新闻发布制度，健全“处早、处小、处快”的网络舆情处理机制；创新信访工作体制机制，深入推行重大决策社会稳定风险评估工作；严格食品药品安全监管，确保市民饮食和用药安全。

　　三是行利民之事。大力实施教育立市战略，办好人民满意的教育。按照“大统筹、大投入、大发展、大成效”的要求加快教育改革发展，继续压缩行政经费的8％用于教育，多渠道筹措教育经费，确保教育投入占生产总值的比重保持在4％以上，推进教育优先发展、优质发展。启动学前教育三年提升行动计划，深入实施教育“9+3”计划，新建或改扩建公办幼儿园8所、城镇义务教育学校8所，建设农村寄宿制学校学生生活用房4.6万平方米，建成国际化学校1所、启动3所，引进优质学校（优质教育管理平台）30个以上，加快建设贵州（清镇）职教城，全面实施职业院校“双证书”制度。提升医疗服务水平。实施好城乡居民大病医疗保险试点工作；健全双向转诊制度；加快建设市儿童医院和市公共卫生救治中心，建成市一医一分院；标准化改扩建乡镇卫生院10个、村卫生室50个，进一步完善基层医疗卫生服务网络。大力发展文体事业。全面开展国家公共文化服务体系示范区创建工作，启动16个公共文化重点工程项目和24个公共文化服务品牌项目建设，促进基本公共文化服务均等化。启动实施贵州文化广场项目，加快推进奥体中心二期、市档案馆和阳明文化产业园、“两带两园一基地”文化旅游功能区建设，建成国际中华文化研修园，办好贵阳观山湖国际马拉松赛等重大赛事，继续推进农村广播电视“村村通”工程，努力满足群众精神文化需求。

　　四是兴便民之举。按照把职能转向基层、让服务贴近群众的要求，继续深化城市基层管理体制改革，积极推行社保、民政、城市管理等职能和部分审批权限下放或委托到社区，提高社区的统筹能力。以“菜篮子”、“米袋子”、“果盘子”等工程为重点，加强基地建设，畅通流通体系，升级改造生鲜超市和农贸市场，建立以标准化农贸市场为主体，生鲜超市、社区生鲜直销店为重要补充的农产品流通网络，切实控制居民生活消费品价格和收费标准上涨幅度，保障农产品供给和促进物价稳定。进一步完善提升“12319”公共服务平台，打造城市便民服务品牌。

　　深入开展双拥创建活动，争创第七次全国双拥模范城，巩固军政、军民团结的大好局面。做好第三次经济普查工作和“十三五”规划前期工作。继续推进民族宗教、外事侨务、对台、移民、史志、档案、老龄、残疾人、国家安全等事业发展，支持国防建设和法院、检察院工作，支持工青妇、关工委、计生协会等组织做好工作。

　　三、切实加强政府自身建设

　　始终秉持实干兴市、实干富民的理念，进一步增强省会意识、机遇意识、担当意识，认真贯彻落实十八届中央纪委三次全会精神，扎实开展党的群众路线教育实践活动，按照“照镜子、正衣冠、洗洗澡、治治病”的总要求，以“三实三同”为统领，以问题为导向，深化“三找”主题大讨论活动，全面转作风、强执行、提效能、优服务，切实推进效能政府、法治政府、廉洁政府、俭朴政府建设。

　　（一）同频共振，提升政府执行力。“一分部署、九分落实”，坚决贯彻执行市委的各项决策部署，做到市委有部署、政府有落实，市委有要求、政府有行动，不观望、不争论、不畏难，埋头苦干、少说多干，把各项工作抓实抓细抓到位。坚决按照“动起来、干起来、统起来、严起来”的要求强化统筹协调，发扬“钉钉子”精神，以不达目的、誓不罢休的劲头打好“攻坚战”，以踏石留印、抓铁有痕的作风啃下“硬骨头”，在全市上下形成抓落实、促发展的良好氛围。

　　（二）转变职能，提升政府服务力。全面正确履行政府职能，把工作重心和主要精力放在创造良好发展环境、提供优质公共服务、维护社会公平正义上来，寓管理于服务之中。健全完善直接联系服务群众的工作机制，扎实开展“三进三服务”活动，大力推行领导在一线指挥、干部在一线工作、问题在一线解决、作风在一线转变的“一线工作法”，着力解决联系服务群众的“最后一公里”问题，多谋民生之利，多解群众之忧。始终坚持权为民所用、利为民所谋，努力用政府权力的“减法”换取市场活力的“加法”，用政府的工作效率换取企业的发展效益，用干部的辛苦指数换取群众的幸福指数。

　　（三）依法行政，提升政府公信力。坚持民主集中制，完善科学民主决策机制，进一步拓宽社情民意反映渠道，做到问政于民、问需于民、问计于民，提高决策科学化、民主化、法制化水平。强化法治思维，严格按照法定权限和法定程序行使权力、履行职责，自觉接受市人大及其常委会的法律监督和工作监督，主动接受市政协的民主监督，健全重大行政决策事项向听取人大报告和听取、政协意见制度，认真办理人大代表建议和政协提案。自觉接受舆论监督、社会监督，加强审计监督，强化政务公开，增强政务透明度，确保政府的权力在阳光下运行。

　　（四）清正廉洁，提升政府表率力。全面落实党风廉政建设责任制和中央、省委、市委各项规定，切实加强政府廉政建设。严格执行“约法三章”，政府性楼堂馆所一律不新建，财政供养人员只减不增，“三公”经费只减不增。坚决反对和纠正“四风”，着力整治“庸懒慢浮贪”，严肃查处“吃拿卡要”和不作为、慢作为、乱作为等行为。始终敬仰党的事业、敬畏手中的权力、敬重服务的人民，始终战战兢兢、如履薄冰、为政清廉，始终保持反腐的高压态势，坚决查处各类违纪违法案件，做到干部清正、政府清廉、政治清明，以“为民务实清廉”的良好政风引领社会新风。

　　各位代表，宏伟的蓝图激励着我们，群众的期盼鞭策着我们！让我们紧密团结在以习近平同志为总书记的党中央周围，在省委、省政府和市委的坚强领导下，牢记“作表率、走前列、做贡献”的历史使命，同心同德、同甘共苦、同频共振，进一步弘扬贵阳精神、增强贵阳自信、保持贵阳定力、汇聚贵阳力量，毫不动摇地实施创新驱动战略，为打造贵阳发展升级版、推进全国生态文明示范城市建设、率先在全省实现全面小康而努力奋斗！
